# Music Intervention for Pain Control in the Pediatric Population: A Systematic Review and Meta-Analysis

**DOI:** 10.3390/jcm11040991

**Published:** 2022-02-14

**Authors:** Berne Ting, Chia-Lin Tsai, Wei-Ti Hsu, Mei-Ling Shen, Ping-Tao Tseng, Daniel Tzu-Li Chen, Kuan-Pin Su, Li Jingling

**Affiliations:** 1 Ph.D. Program for Aging, College of Medicine, China Medical University, Taichung 404, Taiwan; berne.ting@gmail.com; 2 Mind-Body Interface Laboratory (MBI-Lab), China Medical University Hospital, Taichung 404, Taiwan; u9702602@cmu.edu.tw (C.-L.T.); u105023415@cmu.edu.tw (D.T.-L.C.); 3 Graduate Institute of Biomedical Sciences, College of Medicine, China Medical University, Taichung 404, Taiwan; u108305203@cmu.edu.tw; 4 Department of Anesthesiology, China Medical University Hospital, Taichung 404, Taiwan; 5 Taichung Tzu-Chi Hospital, Taichung 427, Taiwan; a8301058@yahoo.com.tw; 6 Department of Psychology, College of Medical and Health Science, Asia University, Taichung 413, Taiwan; ducktseng@gmail.com; 7 Prospect Clinic for Otorhinolaryngology & Neurology, Kaohsiung 811, Taiwan; 8 Institute of Biomedical Sciences, National Sun Yat-sen University, Kaohsiung 804, Taiwan; 9 M.D.-Ph.D. Program, College of Medicine, China Medical University, Taichung 404, Taiwan; 10 School of Chinese Medicine, College of Chinese Medicine, China Medical University, Taichung 404, Taiwan; 11 An-Nan Hospital, China Medical University, Tainan 709, Taiwan

**Keywords:** children, infant, music intervention, pain control

## Abstract

Music intervention (MI) has been applied as an effective adjunctive treatment for pain control in various clinical settings. However, no meta-analysis has yet been published on the analgesic effects of MI in infants and children. We performed a systematic review of PubMed, EMBASE, Web of Science, and Cochrane Library databases to identify randomized controlled trials (RCTs) with the keywords “pain” AND “music therapy” from inception to January 2022. Primary outcomes were pain intensity and vital signs. Standardized mean difference (SMD) values and the corresponding 95% confidence intervals (CIs) were computed using a random effect model. Subgroup analyses with age groups, types of pain, and music styles were conducted. A total of 38 RCTs involving 5601 participants met the selection criteria. MI significantly decreased the pain levels (SMD = −0.57, *p* < 0.001), both in the newborn group (*p* = 0.007) and in the infant/children group (*p* < 0.001). MI significantly reduced heart rate (SMD = −0.50, *p* < 0.001) and respiratory rate (SMD = −0.60, *p* = 0.002) and increased peripheral capillary oxygen saturation (SMD = 0.44, *p* < 0.001). In subgroup analyses of types of pain, MI had significant effects on prick pain (*p* = 0.003), chronic and procedural pain (*p* < 0.001), and postoperative pain (*p* = 0.018). As for music styles, significant analgesic effects were observed for classical music (*p* < 0.001), kids’ music (*p* < 0.001), and pop music (*p* = 0.001), but not for world music (*p* = 0.196), special composition (*p* = 0.092), and multiple music combinations (*p* = 0.420). In conclusion, our analysis provides supportive evidence about the efficacy of MI, especially classical, kids’, and pop music, in controlling prick, procedural, and postoperative pain in the pediatric population.

## 1. Introduction

Pain has significant impacts on both physical and psychological well-being of newborns, infants, and children [1]. Pain may lead to fear, anxiety, depression, and behavioral and cognitive changes [2]. Moreover, several physiological responses, such as plasma cortisol levels, oxygen saturation, heart rate, and respiratory rate, may also be triggered by the unpleasant experience or emotional state of pain [3]. Pharmacotherapy is currently the main treatment for pain relief, with, e.g., non-steroidal anti-inflammatory drugs (NSAIDs). However, it has several adverse effects especially for young populations [4]. Therefore, several non-pharmacological interventions have been applied as adjunctive treatments for pain control in the pediatric population [5], including distraction via taste (e.g., glucose), tactile (e.g., hugs, massage, and acupuncture), auditory (e.g., mother’s voice, imitating the sound of the uterus, and heartbeats), or visual (e.g., cartoons and pictures) stimuli, nutritional supplementation [6], and music interventions.

Music intervention (MI), including music therapy and music treatment [7,8], refers to a non-invasive systematic interventional process in which music is delivered by therapist or medical personnel to improve patients’ health outcomes [9,10,11]. MI facilitates a sense of control in patients [12] and provides mental distraction [8,13], emotional smoothness, and relaxation, which have been found to have pain relief effects.

The effects of MI in reducing pain have been extensively studied in adults, including in 13 recent meta-analyses published from 2000 to 2021 [7,14,15,16,17,18,19,20,21,22,23,24,25]. However, a comprehensive meta-analysis of MI effects in infants or children is still lacking. Therefore, this current meta-analysis mainly focused on clinical trials of MI in newborn infants and children.

## 2. Materials and Methods

### 2.1. Search Strategy and Selection Criteria

Four databases, PubMed, Embase, Web of Science, and Cochrane library, were used to identify studies about the effectiveness of MI in children, from inception date to January 2022. The combination of “pain” and “music therapy” was used to search potential papers in these databases. Two authors, independently, (Hsu and Shen) searched and screened the relevant literature. Firstly, EndNote X8 software was utilized to delete duplicates and non-pediatric literature. After that, the titles and abstracts of all identified articles were assessed for eligibility considering the following inclusion criteria: (1) randomized controlled trials; (2) intervention group receiving MI that included all three factors of music (i.e., rhythm, melody, and harmony); (3) outcome assessments included pain measures; (4) age of all participants was less than 18 years. The exclusion criteria were as follows: (1) reviews, protocols, conference papers, case reports, letters, or editorials; (2) MI was administered with other types of therapy or was a part of complementary and alternative therapy; (3) the control group received any components of music, i.e., rhythm, melody, and harmony; (4) studies that did not provide information for meta-analysis. Finally, the full texts of the identified articles were assessed for meta-analysis by three independent authors (Shen, Tsai, and Ting). If there were any disagreements about the inclusion of the studies, online meetings were convened with the advisors (Jingling and Su) for resolving the conflicts.

### 2.2. Data Extraction

We developed a form to extract the suitable data including the following details: (1) characteristics of the papers (authors, publication year, and country); (2) characteristics of the participants (condition, kinds of pain); (3) study design and methodological quality (random allocation, blinding, selection process of participants, loss to follow-up); (4) MIs (MI method, music style, and use of equipment); (5) outcome measures and statistical data (kind of pain scales and results of pain scores, vital signs, sample size, mean age, and sex ratio). Three authors, independently, (Hsu, Shen, and Ting) extracted the data, and disagreements were resolved by discussing with the other two authors (Jingling and Su).

### 2.3. Assessment of Risk of Bias in the Included Studies

The risk of bias in the included studies was assessed based on the Cochrane Collaboration’s tool [26] by three authors, independently (Chen, Shen, and Tsai). The eight special items for assessing quality and bias judgment were: (1) Random sequence generation, (2) Allocation concealment, (3) Blinding of the participants, (4) Blinding of the personnel who administered the intervention, (5) Blinding of outcome assessment, (6) Incomplete outcome data, (7) Selective reporting, and (8) Other bias. Each item was rated as “low risk,” “unclear risk,” or “high risk” for bias according to the content of the article. In this study, blindness was assessed separately for participants and personnel because the intervention administrators and the researchers might not be the same. We set that “Selective reporting” would evaluate whether the clinical trial was registered. Further, “Other bias” would check the details of the conflict of interests or funding sources. Any disagreements of the results were resolved by discussing with the other two authors (Jingling and Su).

### 2.4. Statistical Analysis

All outcome data included in this meta-analysis were continuous data and were analyzed using the standardized mean differences (SMD) with 95% confidence intervals (CIs). The primary outcome was the pain scores after the intervention for the MI and the control groups. If the outcomes were measured for several points in time, the shortest time point (e.g., five minutes) after the intervention was chosen. If there were multiple assessments, the self-report of the child was chosen for the primary outcome. The secondary outcomes were the vital signs. If standard deviation (SD) or 95% CI were not reported in the original articles, data were estimated from medians, interquartile range (IQR), range, standard errors, t values, or *p* values. The random effect model was used to estimate the pooled effect size [27]. The interpretation of the effect size based on Cohen’s guidelines is as follows: effect size = 0.2 is considered a ‘small’ effect size, 0.5 represents a ‘medium’ effect size, and 0.8 a ‘large’ effect size [28]. All *p*-values were two-sided, and 0.050 was considered statistically significant. Each analysis was evaluated by statistical heterogeneity using I-square (*I*^2^) statistics. A *p*-value less than 0.10 for the *I*^2^ test indicated significant heterogeneity [27]. The potential publication bias was investigated by a funnel plot and Egger’s regression asymmetry analysis [29].

### 2.5. Subgroup Analysis

We found that many of the included studies were targeted at premature babies. Therefore, we further categorized them into three different age subgroups according to the majority of the participants in that study: newborn (less than 3 months or 48 weeks of gestational age), infant and children (3 months to 12 years of age), and adolescent (12 to 18 years of age). On the other hand, the type of music was categorized into six subgroups by a professional musician (Ting) according to the following criteria: (1) Classical music: music written in a Western musical tradition, usually using an established form (for example, a symphony) is generally considered to be serious and to have a lasting value [30]. (2) Children’s music or kids’ music: music composed and performed for children. (3) World music: folk music from around the world. (4) Pop music: a genre of popular music that originated in its modern form during the mid-1950s in the United States and the United Kingdom. The terms popular music and pop music are often used interchangeably, although the former describes all music that is popular and includes many disparate styles. (5) Special composition: music composition for specific objectives; here, it refers to those studies where the researchers designed a special piece of music for pain relief. (6) Multiple Combinations: a simultaneous combination of two or more types of music.

We performed subgroup analyses to investigate the potential heterogeneity of the included studies. The subgroup variables included age groups (newborns, infants, children, and adolescent), pain types (chronic pain, procedural pain, postoperative pain, and prick pain), music styles (classical music, kids’ music, world music, pop music, special composition, multiple combinations), type of equipment (headphone, earphone, speaker, and live performance), and rating sources (other rating by a parent or investigator and self-rating by the children). Sensitivity analyses were conducted to assess the robustness of the findings by excluding the studies with high risk for randomization and allocation concealment. The Comprehensive Meta-Analysis software, version 3, (Biostat, Englewood, NJ, USA) was used to process the statistical data from all included studies.

## 3. Results

### 3.1. Identification of Eligible Studies

**Figure 1:**
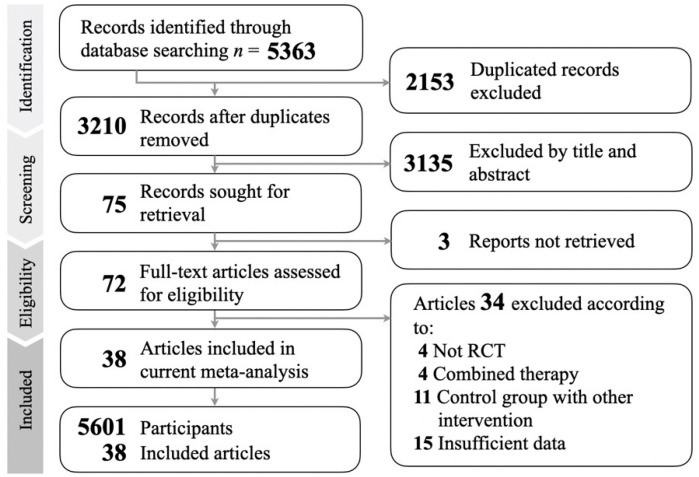
Flow chart of the selection strategy and inclusion and exclusion criteria.

Figure 1 shows the result of our screening process. We identified 5363 articles with our searching strategy. Duplicate articles (*n* = 2153) were excluded. The abstracts of articles that did not match the selection criteria (*n* = 3135) were also excluded. Finally, 38 articles out of 72 available full-text articles were included in this meta-analysis. The details of the excluded 34 articles are presented in Supplementary File S1.

### 3.2. Study Characteristics and Patient Populations

A total of 5601 participants in 38 articles, published between 2006 and 2022, were included (Table 1). Among all articles, eight articles were conducted in America [31,32,33,34,35,36,37], 14 in Europe [38,39,40,41,42,43,44,45,46,47,48,49,50,51], 13 in Asia [52,53,54,55,56,57,58,59,60,61,62,63,64,65], and three in Africa [11,66,67]. Most studies (*n* = 33) had a parallel randomized controlled design, and five studies [39,45,48,53,62] had a cross-over randomized controlled design. The sample size in each article ranged from 20 to 3095 participants. The mean age of the participants ranged from a few days of birth to teenage years. To be specific, two articles included adolescents [43,52], 12 newborns [31,39,41,48,49,53,56,60,61,62,64,65], and 24 infants and children [11,32,33,34,35,36,37,38,42,44,45,46,47,50,51,54,55,57,58,59,63,66,67]. On the other hand, 29 studies were conducted in the hospital setting [11,32,33,34,36,38,39,40,41,44,45,46,47,48,49,50,51,53,54,55,56,58,59,60,61,62,64,65,66,67], and 9 in the clinic setting [31,35,37,42,43,51,52,57,63]. As for the type of pain, 18 articles examined participants with prick pain (heel lance, intravenous (IV) insertion, immunization, acupuncture, and lumbar puncture) [33,35,37,39,41,43,48,51,53,54,56,58,59,60,62,63,64,65], 8 participants with procedural pain (emergency procedural, retinopathy of prematurity (ROP), wound care, peripherally inserted central catheter (PICC), orthodontic procedure, and continuous positive airway pressure (C-PAP)) [11,31,32,49,50,52,61,67], 4 participants with chronic pain (rehabilitation, illness pressure) [42,45,57,66], and 8 participants with postoperative pain [34,36,38,40,44,46,47,55]. Among all 38 studies, the participants listened to classical music in 11 articles [32,34,39,40,41,42,44,46,62,64,66], to kids’ music in 8 articles [31,36,37,38,45,49,61,65], to world music in 3 articles [54,56,60], to pop music in 3 articles [51,57,63], to special composition in 5 articles [11,35,47,48,59], and to multiple combinations of music in 4 articles [33,43,50,67]. Additionally, in one article [53], two groups of participants listened to either kids’ music or world music, and thus this article was separated into two datasets. As for the listening instruments, 9 articles used headphones [32,34,35,36,38,44,53,63,65], 4 used earphones [46,54,55,59], 19 used speakers [11,31,33,39,40,41,42,47,48,49,51,56,58,60,61,62,64,66], and 4 used live performance [37,45,50,67]. In one article [43], participants listened to music using either headphones or speakers, and thus this study was separated into two datasets. A total of 14 different pain rating scales were used in the meta-analysis, consisting of Likert pain scales (Wong–Baker Scale (WBS), Visual Analog Scale (VAS), Numeric Rating Scale (NRS), Faces Pain Scale (FPS), and Faces Pain Scale-Revised (FPS-R)) and structured pain scales (Neonatal Pain, Agitation, and Sedation Scale (N-PASS), Premature Infant Pain Profile (PIPP), COMFORT behavior scale (COMFORT-B), Children’s Hospital of Eastern Ontario Pain Scale (CHEOPS), Neonatal Facial Coding System (NFCS), Neonatal Infant Pain Scale (NIPS), Child–Adult Medical Procedure Interaction Scale Revised (CAMPIS-R), Face, Legs, Activity, Cry, Consolability scale (FLACC), and Oucher Pain Scale (OPS)). Two articles included multiple intervention groups, and thus more comparisons could be carried out. Finally, 40 datasets (MI vs. control) were extracted for meta-analysis from the 38 articles.

### 3.3. Quality of the Included Articles

As for the quality of the 38 articles, only 3 articles had a high risk of selection bias [34,37,65], and one article had a high risk of attrition bias [62]. Due to the nature of MI, most of the included articles had high a risk of performance bias, but five articles had a low risk, by blinding both the participants and the researchers [32,38,44,46,65]. As for detection bias, 15 articles [31,37,38,44,45,46,48,49,53,56,57,58,60,65,67] had a low risk by blinding the researchers. The graph and summary table of risk of bias are presented in Supplementary File S2.

### 3.4. Primary Outcome of MI

**Figure 2:**
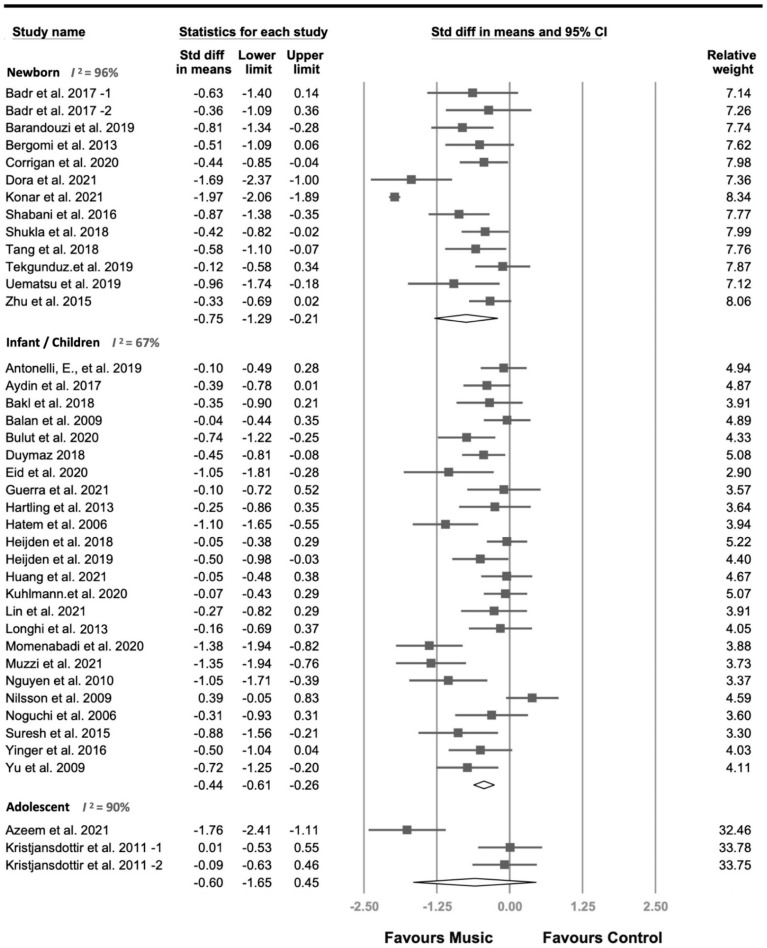
Forest plot for the effects of music on pain score.

**Figure 3:**
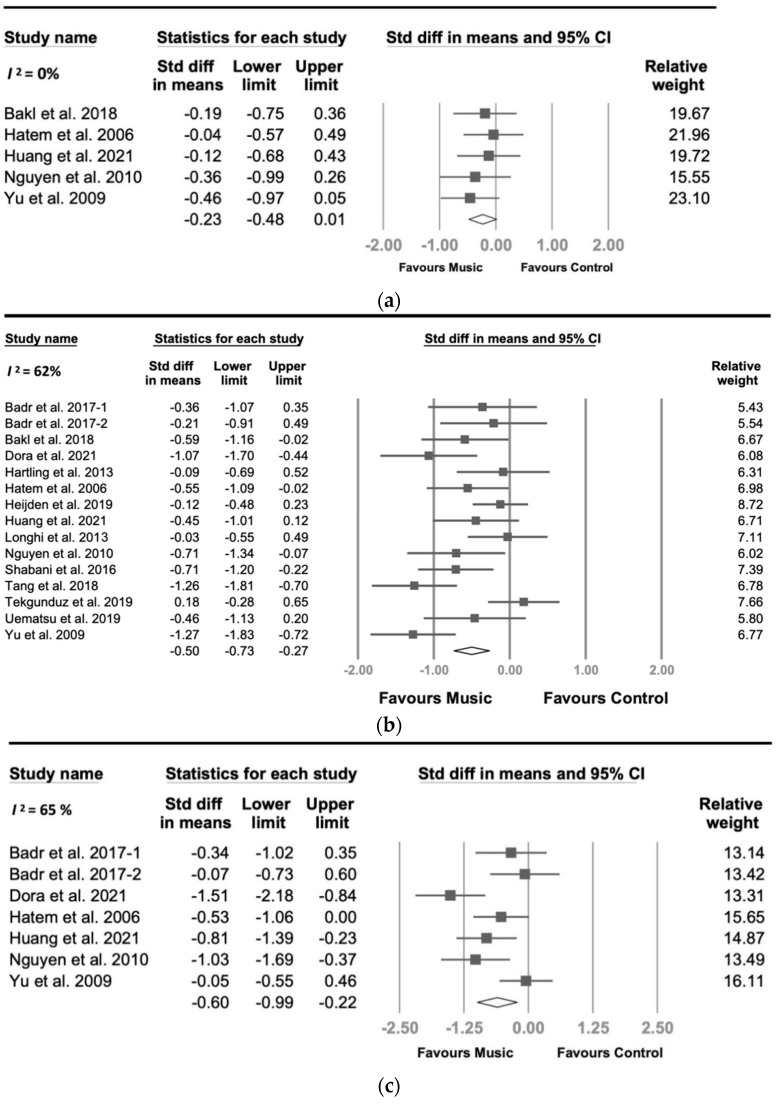

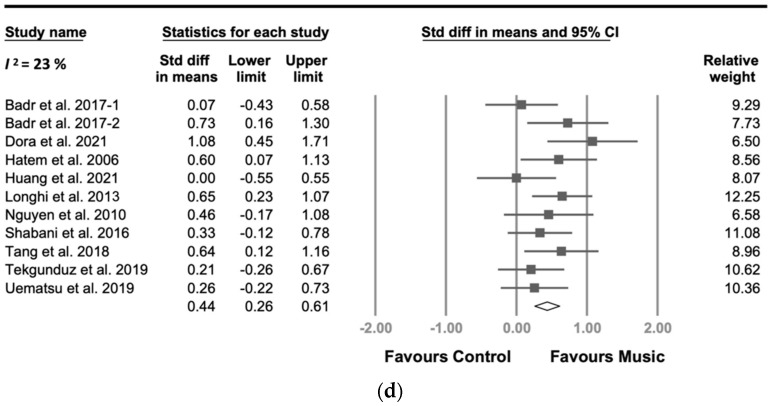
Forest plots for the effects of music on vital signs. (**a**) Blood pressure, (**b**) Heart rate, (**c**) Respiratory rate, and (**d**) Peripheral capillary oxygen saturation (SpO_2_).

Figure 2 shows the primary results of our extracted datasets (*n* = 40), involving 5601 participants. We found that MI significantly decreased the pain score (SMD = −0.57, 95% CI = −0.87 to −0.27, *p* < 0.001), but with significantly heterogeneity (*I*^2^ = 95%, *p* for *I*^2^ < 0.001). Therefore, subgroup analysis on age was carried out and is described in Figure 3. The effect size (−0.57) indicated that MIs had a medium effect on pain control. Analysis based on groups of age revealed that music decreased pain significantly in both newborns (*k* = 13, *n* = 3774, SMD = −0.75, 95% CI = −1.29 to −0.21, *p* = 0.007) and infants and children (*k* = 24, *n* = 1659, SMD = −0.44, 95% CI = −0.61 to −0.26, *p* < 0.001). Meanwhile, music showed no evidence of a pain-relieving effect in adolescents (*p* = 0.264). This is likely due to the small number of studies in adolescents.

### 3.5. Secondary Outcome of MI

Figure 3 describes the effect size findings for vital signs, including blood pressure (BP), heart rate (HR), respiration rate (RR), and peripheral capillary oxygen saturation (SpO_2_). BP was recorded in five datasets with 279 participants and was not significantly affected (*p* = 0.063) by MI for pain relief. The analysis of 15 datasets involving 743 participants indicated that MI lowered HR by 0.50 units (95% CI −0.73 to −0.27, *p* < 0.001), with significant heterogeneity (*I*^2^ = 62%, *p* for *I*^2^ < 0.001). Seven datasets with 315 participants revealed that MI showed a medium effect on RR, but with significant heterogeneity (SMD = −0.60, 95% CI = −0.99 to −0.22, *p* = 0.002, *I*^2^ = 65%, *p* for *I*^2^ = 0.010). SpO_2_ was measured in 11 datasets, involving 469 participants, and a small to medium but significant effect (SMD = 0.44, 95% CI = 0.26 to 0.61, *p* < 0.001, *I*^2^ = 23%) was observed. The positive SMD indicated that SpO_2_ was higher in the music group than in the control group. The results of vital signs revealed that MIs showed statistically significant effects in decreasing HR and RR and increasing SpO_2_ in children in painful conditions, while not influencing BP.

### 3.6. Subgroup Analyses

Table 2 shows the effect of MI on pain by collapsing data from all age groups in subgroup analyses. In general, MI had a significantly superior effect in decreasing chronic and procedural pain (SMD = −0.64, *p* < 0.001), postoperative pain (SMD = −0.49, *p* = 0.018), and prick pain (SMD = −0.66, *p* = 0.003). As for the music styles, the results revealed that MI produced a significantly superior effect on pain release when classical music, kids’ music, and pop music were provided. To be specific, classical music showed the largest effect size (SMD = −0.71, *p* < 0.001) in reducing pain in children. On the contrary, the results showed the limited effects of world music, special composition, and multiple combinations of music. In the subgroup analysis by type of equipment, MI exhibited a significantly superior effect when music was delivered by headphones and earphones (SMD = −0.49, *p* < 0.001) and speaker (SMD = −0.63, *p* = 0.005) compared to the control groups. There was a limited effect of music delivered during live performance.

We included studies that measured pain intensity by using both other rating methods (PIPP, NIPS, NFCS, Comfort-B, CAMPIS-R, CHEOPS, N-PASS, and FLACC) and self-rating methods (VAS, WBS, FPS, FPS-R, NRS, and OPS). A similar effect of music in reducing pain scores was found when using both other rating methods (19 data, SMD = −1.04, *p* = 0.017) and self-rating methods (21 data, SMD = −0.76, *p* < 0.001). These results revealed that MIs are reliably effective in reducing pain in children, regardless of assessing methods.

### 3.7. Sensitivity Analysis

Sensitivity analysis was conducted on selection bias by including only high-quality studies on randomization and allocation concealment [11,32,33,36,42,44,46,56,60,62,63,64,67] and revealed a significantly median to large effect size (*k* = 13, *n* = 4022, SMD = −0.62, 95% CI = −1.20 to −0.04, *p* = 0.037, *I*^2^ = 97%). The result of sensitivity analysis showed a similar effect size as that of the main analysis (SMD = −0.57, *p* < 0.001), indicating that the main result was robust.

### 3.8. Evaluation of Publication Bias

**Figure 4:**
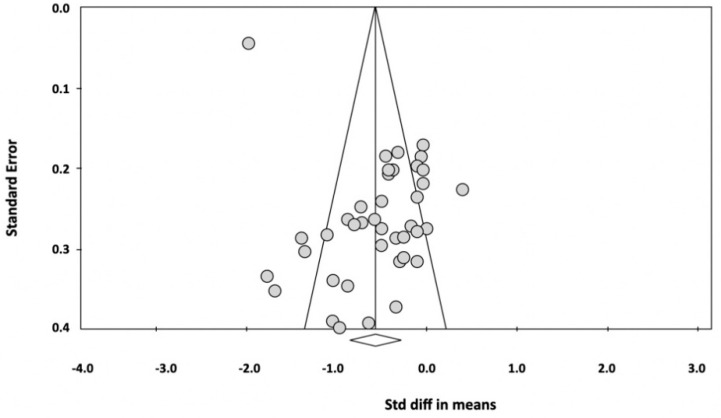
Funnel plot of standard difference, with means on the X-axis and standard error on the Y-axis for the effect of music on pain release.

Publication bias easily occurs in studies with small sample sizes, referring to the phenomenon that studies with significant results are more likely to be published than those reporting nonsignificant conclusions [68]. The funnel plot of standard errors by SMD was assessed according to its symmetry, and the results are presented in Figure 4. The results showed asymmetry, with a blank area in the lower right region of the funnel plot indicating the absence of articles with small sample size and non-significant results for publication [69]. Meanwhile, a significant publication bias was founded by Egger’s regression test (*t* = 7.53, df = 38, 2-tailed *p* < 0.001).

## 4. Discussion

To our knowledge, this is the first meta-analysis focused on pain control by MI in newborn babies. Although several past studies focused on this topic, they were not meta-analyses. For instance, O’Toole et al. and González-Martín-Moreno et al. reviewed the effects of MIs on pain in infants and children [70,71]. Another previous study investigated the effect of MI on pain in children; however, they did not find consistent outcome measures for a meta-analysis [72]. Meanwhile, a meta-analysis probed the effect of music in children but focused on anxiety and quality of life in children rather than on pain [73]. Therefore, our study provides a comprehensive summary of current RCTs on pain control by MI in pediatric populations.

Our main finding is that MI can decrease pain levels and stabilize HR, SPO_2_, and RR in the pediatric population. MI provides a significant analgesic effect in infants and children, especially in newborn babies. As for the different types of pain, we found that MI showed positive results for prick pain, procedure pain, and postoperative pain. Our findings are consistent with some previous meta-analyses that reported significant effects in pain control, such as pain in surgery [74,75] and postoperative pain [20,76,77]. Among all the music styles we investigated, classical music, kids’ music, and pop music had the greatest impact on alleviating pain in children. The effect of MI can be delivered by headphones, earphones, or speakers. The effect of pain control may be linked to individual music preferences. Besides, specific features such as rhythm and harmony and the use of specific instruments also seem important for anxiety and pain reduction [47]. However, we cannot expect the development of music preference in infants and children is completely established. In general, MI can robustly provide pain relief across various conditions.

Music provides a relaxing atmosphere for newborns via the auditory system, which is fully developed before birth. To be specific, the auditory perception of a fetus is developed at about the 25th week of gestation [78], and objective indicators of normal cognitive ability, including auditory perception, can be measured in the first few days after birth [79]. Therefore, music can be appreciated even by newborn babies, which indicates auditory access may be a key to pain relief [80]. On top of that, MI delivers rhythm, melody, and harmony that may smooth emotion and lead to relaxation [12]. Compared to other non-invasive and non-pharmacological pain relief interventions, such as sucrose and massage, MI is recommended for its convenience and effectiveness. For instance, specialists are needed to massage, and other artificial products are taken when using sucrose as an analgesic intervention. By contrast, no specific material or faculty is needed for MI. It could be argued that a placebo effect is beneficial anyway [81], in this instance reducing pain. However, the subgroup analysis did show a statistically significant pain-reducing effect. The placebo effect could be limited, as the studies relied on both self-reporting and observational rating by others in pediatric populations, indicating objective results in our study. The effects on pain control may be associated with distraction provided by music [8,13]. In fact, a meta-analysis by Richard-Lalonde et al. investigating the pain control effect of music in an adult mixed population admitted to the ICU also showed a significant positive result [82]. More importantly, international guidelines for ICU care recommend offering music to reduce pain and strongly recommend further research on non-pharmacological interventions [83]. However, it currently still lacks related guidelines for infants and children. After all, MI is still recommended to improve short-term outcomes in NICU for premature infants, for its effectiveness and safety.

We further revealed that the analgesic effect of MI can be achieved by various music styles (Table 2). Interestingly, monotype of classical music, kids’ music, and pop music showed better effects in reducing pain, while the combination of multiple music types was not effective. Those studies with multi-style combinations of music [33,43,50,72] showed inconsistent results, probably due to the mixture of emotional reactions induced by different music styles [48]. For instance, music with low bass and low beats can stimulate the limbic system of the brain and thus may affect emotional regulation [11,48,84]. Further, relaxing music can reduce both HR and BP, while music with a fast beat may have the opposite effect [73]. Therefore, a mixture of music types may have opposite effects on emotional regulation, leading to insignificant results. Another interesting finding is that several ways to deliver music, i.e., the use of headphones, earphones, and speaker, can significantly reduce pain, except for live performance. We considered that this may be caused by components of live performance other than music that dilute the effect of music [50]. In summary, a consistent music style and pure auditory inputs may be crucial for the success of MI in pain relief. We then also suggest that optimal sound in MI for the pediatric population, such as specific basses, rhythms, and tones, might be worthy of testing in the future.

Our study has a number of limitations and offers suggestions for issues that could be addressed in future research. Firstly, finding results with the employed search strategy might be insufficient. For instance, only two articles with three datasets were included for adolescent participants. Future studies may focus on critical factors that may contribute to the analgesic effect of MI specifically in this age group. Secondly, many studies were not double-blinded, which is one of the fundamental challenges of MI. Whether participants or experimenters are aware of the effect of music during the intervention can be considered in future studies to reduce the placebo effect. Thirdly, a high heterogeneity was shown even in the subgroup analysis (Figure 3 and Table 2). Therefore, it is possible that some unnoticed factors may have not been categorized in our current analysis, and these factors may play an essential role in the effect of MI on pain. For instance, adolescents might have developed their preference of music style, and providing music that was unappealing to them may have reduced the effectiveness of the intervention [33]. Alternatively, the high heterogeneity might reveal the heterogeneity of studies we included in the meta-analysis. However, we considered that 40 comparisons in our meta-analysis would provide a reliable estimation of the analgesic effect of MI. Lastly, the subject number (total *n* = 3095) of an included study [56] was much larger than those of other included studies, which may have influenced the outcomes of our analyses. To this end, software CAM automatically provided a relative weight for correction. Therefore, the results should also be reliable.

## 5. Conclusions

Our meta-analysis of 38 RCT articles with a total of 5601 participants provides evidence supporting the thesis that MI releases pain in both psychological and physiological domains. A consistent music style and a pure auditory experience might be important in MI for pain. MI is an appropriate, low-stress, and safe non-pharmacological treatment for clinical pain relief in the pediatric population.

## Figures and Tables

**Table 1:** Characteristics of the recruited 38 studies.

Authors & Year	Journal	Country	Study Design	Comparison	Participants, No.	Age, Mean (SD), y/m/w/d	Age Group	Specialization	Setting	Condition	Pain Types	Type/Genres/Titles of Music	Equip	Outcome Measure/Assessment Tools
Antonelli et al., 2019	Wolters kluwer	Italy	Parallel RCT	Music Control	57 48	8.20 (3.3), y 8.30 (4.1), y	Infant/Children	Emergency	In	ER pressure	Chronic/Procedural	Multiple Combinations	Live	WBS
Aydin et al., 2017	Applied nursing research	Turkey	Parallel RCT	Music Control	50 50	8.68 (2.2), y 9.20 (2.6), y	Infant/Children	Pain clinic	Out	IV insertion	Prick pain	Pop music	SPK	WBS
Azeem et al., 2021	Cochrane Central Register of Controlled Trials	Pakistan	Parallel RCT	Music Control	25 25	12–16, y ♫	Adolescent	Dentistry	Out	Postop pain	Chronic/Procedural	--	--	VAS
Badr et al., 2017	Acta Paediatr	Lebanon	Cross-over RCT	Music Control	42	34.83 (1.3), GA, w	Newborn	Pediatrics	In	Heel Lance	Prick pain	Kids music/ World music	HDP	N-PASS HR, RR, SpO2
Bakı et al., 2018	Medical Principles and Practice	Turkey	Parallel RCT	Music Control	25 25	6.60 (2.95), y 6.24 (3.38), y	Infant/Children	General surgery	In	Postop Pain	Postoperative Pain	Kids music	HDP	WBS BP, HR
Balan et al., 2009	Indian Pediatrics	India	Parallel RCT	Music Control	50 50	8.27 (2.05), y 7.42 (2.19), y	Infant/Children	Pain clinic	In	IV insertion	Prick pain	World music	ERP	VAS
Barandouzi et al., 2019	Complementary Therapies in Medicine	Iran	Parallel RCT	Music Control	30 30	34.00 (1.41), GA, w 33.86 (1.35), GA, w	Newborn	Pediatrics	In	IV insertion	Prick pain	Kids music	HDP	PIPP
Bergomi et al., 2014	Research and Theory for Nursing Practice	Italy	Cross-over RCT	Music Control	35	37.00 (5.43), GA, w	Newborn	Neonatology	In	Heel Lance	Prick pain	Classical music	SPK	PIPP
Bulut et al., 2020	Journal of perianesthesia nursing	Turkey	Parallel RCT	Music Control	35 35	7.37 (0.91), y 7.20 (0.67), y	Infant/Children	Pediatric surgery	In	Postop Pain	Postoperative Pain	Classical music	SPK	WBS
Corrigan et al., 2020	Perinatol	USA	Parallel RCT	Music Control	43 54	34.80 (0.86), GA, w 34.50 (0.92), GA, w	Newborn	Ophthalmology	Out	ROP	Chronic/Procedural	Kids music	SPK	PIPP
Döra et al., 2021	Pain management nursing	Turkey	Parallel RCT	Music Control	22 22	32–37, w, GA ♫	Newborn	Neonatal ICU	In	Blood collection	Prick pain	Classical music	SPK	PIPP HR, RR, SpO2
Duymaz, 2020	Annals of Clinical and Analytical Medicine	Turkey	Parallel RCT	Music Control	60 60	7.42 (2.40), y 7.60 (2.60), y	Infant/Children	Neurology	Out	Rehabilitation	Chronic/Procedural	Classical music	SPK	WBS
Eid et al., 2020	Burns	Egypt	Parallel RCT	Music Control	15 15	9.83 (1.25), y 9.71 (1.16), y	Infant/Children	Physical therapy	In	Rehabilitation	Chronic/Procedural	Classical music	SPK	VAS
Guerra et al., 2021	Journal of Intensive Care	Canada	Parallel RCT	Music Control	20 20	1.16 (3.5) 2.02 (3.5)	Infant/Children	Pediatric ICU	In	ICU procedure	Chronic/Procedural	Classical music	HDP	FLACC
Hartling et al., 2013	JAMA Pediatrics	Canada	Parallel RCT	Music Control	21 21	64.00 (50.27), m 78.00 (70.24), m	Infant/Children	Pediatrics	In	IV insertion	Prick pain	Multiple Combinations	SPK	FPS-R HR
Hatem et al., 2006	Jornal de Pediatria	Brasil	Parallel RCT	Music Control	61 18	1d-16y ♫	Infant/Children	Cardiology	In	Postop Pain	Postoperative Pain	Classical music	HDP	FPS BP, HR, RR, SpO2
Huang et al., 2021	The Heart Surgery Forum	China	Parallel RCT	Music Control	42 42	4.8 (1.7), y 5.9 (1.7), y	Infant/Children	Pediatric Surgery	In	Postop pain	Postoperative Pain	--	ERP	WBS HR, BP, RR, SpO2
Konar et al., 2021	Journal of Tropical Pediatrics	India	Parallel RCT	Music Control	1546 1549	34.3 (3.1), w 34.5 (3.0), w	Newborn	Neonatology	In	IV insertion	Prick pain	World music	SPK	N-PASS
Kristjánsdóttir et al., 2011	Scandinavian Journal of Caring Sciences	Iceland	Parallel RCT	Music Control	79 39	14.00 (0.18), y	Adolescent	-	Out	Immunization	Prick pain	Multiple Combinations	HDP/SPK	VAS
Kühlmann et al., 2020	Anesthesia and Analgesia	Netherlands	Parallel RCT	Music Control	59 59	6.90 (14.46), m 7.30 (16.89), m	Infant/Children	Pediatric surgery	In	Postop Pain	Postoperative Pain	Classical music	HDP	COMFORT-B
Lin et al., 2021	The Heart Surgery Forum	China	Parallel RCT	Music Control	25 25	7.3 (1.0), y 6.9 (1.2), y	Infant/Children	Pediatric Surgery	Out	Postop chronic pain	Chronic/Procedural	Pop music	SPK	VAS
Longhi et al., 2013	Psychology of Music	UK	Cross-over RCT	Music Control	37	7d-4y ♫	Infant/Children	Pediatrics	In	Illness pressure	Chronic/Procedural	Kids music	Live	CHEOPS HR, SpO2
Momenabadi et al., 2020	Pakistan Journal of Medical and Health Sciences	Iran	Parallel RCT	Music Control	30 30	51.50 (10.52), m 51.33 (8.63), m	Infant/Children	Pediatric	In	IV insertion	Prick pain	--	SPK	OPS
Muzzi et al., 2021	JAMA Otolaryngol Head Neck Surg	Italy	Parallel RCT	Music Control	26 28	4.5 (3.6–6.0), y ♪ 5.2 (3.6–7.0), y ♪	Infant/Children	Otorhinolaryngology	In	Postop pain	Postoperative Pain	Classical music	ERP	WBS, VAS
Nguyen et al., 2010	Journal of Pediatric Oncology Nursing	Vietnam	Parallel RCT	Music Control	20 20	8.80 (1.59), y 9.40 (1.93), y	Infant/Children	Pediatrics	In	Lumbar puncture	Prick pain	Special composition	ERP	NRS BP, HR, RR, SpO2
Nilsson et al., 2009	Paediatric Anaesthesia	Sweden	Parallel RCT	Music Control	40 40	12 (7–16), y ♪ 13.5 (7–16), y ♪	Infant/Children	General surgery	In	Postop Pain	Postoperative Pain	Special composition	SPK	VAS
Noguchi et al., 2006	Journal of Music Therapy	USA	Parallel RCT	Music Control	21 20	4.55 (0.65), y	Infant/Children	Pediatrics	Out	Immunization	Prick pain	Special composition	HDP	FPS
Shabani et al., 2016	Iranian Journal of Nursing and Midwifery Research	Ireland	Cross-over RCT	Music Control	20	29–36, GA, w ♫	Newborn	Neonatology	In	IV insertion	Prick pain	Special composition	SPK	NFCS HR, SpO2
Shukla et al., 2018	Indian Pediatrics	India	Parallel RCT	Music Control	49 51	8.10 (8.21), d 6.50 (4.38), d	Newborn	Pediatrics	In	Heel Lance	Prick pain	World music	SPK	PIPP
Suresh et al., 2015	Pediatric Surgery International	USA	Parallel RCT	Music Control	18 19	10.90 (5.20), y 12.40 (5.34), y	Infant/Children	General surgery	In	Postop Pain	Postoperative Pain	Kids music	HDP	FPS-R
Tang et al., 2018	European Journal of Integrative Medicine	China	Parallel RCT	Music Control	30 30	32.57 (1.76), GA, w 32. 57 (1.83), w	Newborn	Pediatrics	In	PICC	Chronic/Procedural	Kids music	SPK	PIPP HR, SpO2
Tekgündüz et al., 2019	Pain management nursing	Turkey	Parallel RCT	Music Control	35 37	2.00 (1.91), d 2.49 (2.12), d	Newborn	Neonatology	In	C-PAP	Chronic/Procedural	Kids music	SPK	PIPP; NIPS HR, SpO2
Uematsu et al., 2019	Paediatrics and Child Health (Canada)	Japan	Cross-over RCT	Music Control	25	33.80 (1.5), GA, w	Newborn	Neonatology	In	Heel Lance	Prick pain	Classical music	SPK	PIPP HR, SpO2
ven der Heijden et al., 2018	Burns	South Africa	Parallel RCT	Music Control	71 64	24.30 (71.79), m 20.80 (42.04), m	Infant/Children	Intensive care	In	Wound care	Chronic/Procedural	Multiple Combinations	Live	COMFORT-B
ven der Heijden et al., 2019	Journal of pediatric psychology	South Africa	Parallel RCT	Music Control	75 54	7.50 (11.27), y 7.70 (8.81), y	Infant/Children	Emergency	In	ER procedure	Chronic/Procedural	Special composition	SPK	FPS-R HR
Yinger et al., 2016	Journal of Music Therapy	USA	Parallel RCT	Music Control	29 29	48.10 (6.7), m	Infant/Children	Pediatrics	Out	Immunization	Prick pain	Kids music	Live	CAMPIS-R
Yu et al., 2009	International Journal of Nursing Studies	China	Parallel RCT	Music Control	30 30	8.26 (2.83), y 7.87 (3.35), y	Infant/Children	Traditional Chinese medicine	Out	Acupuncture	Prick pain	Pop music	HDP	WBS BP, HR, RR
Zhu et al., 2015	Midwifery	China	Parallel RCT	Music Control	62 61	3.37 (0.58), d 3.11 (0.49), d	Newborn	Neonatology	In	Heel Lance	Prick pain	Classical music	SPK	NIPS

Note. CAMPIS-R, Child-Adult Medical Procedure Interaction Scale-Revised; CHEOPS, Children’s Hospital of Eastern Ontario Pain Scale; COMFORT-B, COMFORT-B score; FPS, Faces pain scale; FPS-R, Faces pain scale-Revised; FLACC, Face Legs Activity Cry Consolability; N-PASS, Neonatal Pain, Agitation and Sedation Scale; NFCS, Neonatal Facial Coding System; NIPS, Neonatal Infant Pain Scale; OPS, Oucher pain scale; PIPP, Premature Infant Pain Profile; VAS, Visual Analogue Scale; WBS, Wong Baker Faces Pain Rating Scale; BP, Blood Pressure; HR, Heart Rate; RR, Respiratory Rate; SPK, speaker; HDP, headphones; ERP, earphones; Live, Live performance; y, years; m, months; w, weeks; GA, gestational age; d, days; ♪, median (range); ♫, range.

**Table 2:** Subgroup analyses of music for pain release.

Intervention/Moderator	*k*	*n*	Effect Size	95% CI-L	95% CI-U	*p* Value	*I*^2^ (%)
Type of Pain							
Chronic/Procedural	12	869	−0.41	−0.64	−0.64	<0.001	63
Postoperative pain	8	571	−0.49	−0.90	−0.08	0.018	81
Prick pain	20	4161	−0.66	−1.10	−0.23	0.003	95
Music style							
Classical music	11	738	−0.71	−1.00	−0.42	<0.001	71
Kids music	9	489	−0.44	−0.62	−0.27	<0.001	0
Multiple combinations	5	400	−0.08	−0.28	0.12	0.420	0
Pop music	3	210	−0.45	−0.73	−0.18	0.001	0
Special composition	5	254	−0.45	−0.97	0.07	0.092	79
World music	4	3316	−0.78	−1.96	0.40	0.196	98
Type of Equipment							
HDP/ERP	15	862	−0.49	−0.73	−0.26	<0.001	63
SPK	20	4357	−0.63	−1.07	−0.19	0.005	96
Live	4	332	−0.15	−0.36	0.06	0.160	0
Other rating/Self-rating							
Other rating	19	4208	−0.57	−1.04	−0.10	0.017	96
Self-rating	21	1393	−0.54	−0.76	−0.32	<0.001	75

Note. *k*, number of studies; *n*, number of participants; CI, confidence interval; *I*^2^, heterogeneity testing; SMD, standardized mean difference; VAS, visual analog scale; SPK, speaker; HDP, headphone; ERP, earphones; Live, Live performance.

## Data Availability

The data presented in this study are available in Google drive (MAShare_2022), at https://drive.google.com/drive/folders/1wSH6vOveKvkCHytCaOIiTrlz1KAziLFh?usp=sharing (accessed on 2 January 2022).

## Supplementary Materials

The following supporting information can be downloaded at: https://www.mdpi.com/article/10.3390/jcm11040991/s1, Supplementary File S1: Reasons for exclusion of 34 articles; Supplementary File S2: Quality of the included studies: (a) Risk of bias graph; (b) Risk of bias summary.
