# Supplementary material for: Music Intervention for Pain Control in the Pediatric Population: A Systematic Review and Meta-Analysis
Source: J Clin Med. 2022 Feb 14;11(4):991. doi: 10.3390/jcm11040991 (PMC8877634; doi:10.3390/jcm11040991)
Supplement: Supplementary file 1 [file jcm-11-00991-s001.zip › jcm-1562346-supplementary.pdf]

Supplementary File S1. Reasons for exclusion of 34 articles.

Authors & Year	Journal/Book	Title	Exclusion Reason
Antonacci et al., 2021	Music Therapy Perspectives	Effects of Guitar Accompaniment Patterns on Hospitalized Infants: A Randomized Controlled Trial	Control group with other interventions
Atak et al.,2021	Journal of PeriAnesthesia Nursing	The Effect of Different Audio Distraction Methods on Children's Postoperative Pain and Anxiety	Control group with other interventions
Bo et al., 2000	Pediatrics	Soothing pain-elicited distress in Chinese neonates.	Insufficient data
Bush et al., 2021	American Journal of Critical Care	Effect of Live Versus Recorded Music on Children Receiving Mechanical Ventilation and Sedation	Control group with other interventions
Butt et al., 2000	Canadian Journal of Nursing Research Archive	Music modulates behaviour of premature infants following heel lance.	Insufficient data
Calcaterra et al., 2014	Pediatric reports	Music benefits on postoperative distress and pain in pediatric day care surgery	Insufficient data
Caprilli et al., 2007	Journal of Developmental & Behavioral Pediatrics	Interactive music as a treatment for pain and stress in children during venipuncture: a randomized prospective study.	Insufficient data
Cardoso et al., 2014	Revista latino-americana de enfermagem	Music and 25% glucose pain relief for the premature infant: a randomized clinical trial.	Control group with other interventions
Clark et al., 2017	Canadian Journal of Music Therapy	Pediatric Palliative Music Therapy: pain, Distress, and Contentment in Children With Developmental Delays Associated With Life-Threatening Severe Neurological Impairment.	Control group with other interventions
Colwell et al., 2013	Journal of Pediatric Nursing	Impact of Music Therapy Interventions (Listening, Composition, Orff-Based) on the Physiological and Psychosocial Behaviors of Hospitalized Children: A Feasibility Study.	Control group with other interventions
Erickson Megel et al., 1998	Issues in comprehensive pediatric nursing	Children's responses to immunizations: lullabies as a distraction.	Insufficient data
Gao et al., 2021	Pain	Effect of combined pharmacological, behavioral, and physical interventions for procedural pain on salivary cortisol and neurobehavioral development in preterm infants: a randomized controlled trial	Combinded therapy
Howard et al., 2020	Acta Paediatrica	No effect of a musical intervention on stress response to venepuncture in a neonatal population	Control group with other interventions
Huang et al., 2021	Journal of Cardiac Surgery	The music video therapy in postoperative analgesia in preschool children after cardiothoracic surgery	Combinded therapy
Koenig et al., 2013	The Journal of Pain	Specific music therapy techniques in the treatment of primary headache disorders in adolescents: A randomized attention-placebo-controlled trial	Control group with other interventions
Melo et al., 2017	Revista brasileira de enfermagem	Non-pharmacological measures in preterm newborns submitted to arterial puncture.	Control group with other interventions
Nelson et al., 2017	Pain management nursing	Relaxation Training and Postoperative Music Therapy for Adolescents Undergoing Spinal Fusion Surgery.	Combinded therapy
Oelkers-Ax et al., 2008	European Journal of Pain	Butterbur root extract and music therapy in the prevention of childhood migraine: an explorative study.	Insufficient data
Perkins et al., 2018	Frontiers in oncology	Roadmap to wellness: Exploring live customized music at the bedside for hospitalized children.	Insufficient data
Polat et al., 2015	Indian Journal of Traditional Knowledge	The effect of therapeutic music on anxiety in children with acute lymphoblastic leukaemia	Insufficient data
Qiu et al., 2017	BMC pediatrics	Effect of combined music and touch intervention on pain response and beta-endorphin and cortisol concentrations in late preterm infants.	Combinded therapy
Rahlin et al., 2009	Pediatric Physical Therapy	Effects of Music on Crying Behavior of Infants and Toddlers During Physical Therapy Intervention.	Not RCT
Rossi et al., 2018	Early human development	Music reduces pain perception in healthy newborns: A comparison between different music tracks and recoded heartbeat.	Insufficient data
Scheufler et al., 2021	Journal of music therapy	Comparing Three Music Therapy Interventions for Anxiety and Relaxation in Youth With Amplified Pain	Insufficient data
Scheufler et al., 2021	Journal of music therapy	Comparing Three Music Therapy Interventions for Anxiety and Relaxation in Youth With Amplified Pain	Control group with other interventions
Schwilling et al., 2015	Acta Paediatrica	Live music reduces stress levels in very low-birthweight infants.	Not RCT
Shah et al., 2017	The Journal of pediatrics	Trial of Music, Sucrose, and Combination Therapy for Pain Relief during Heel Prick Procedures in Neonates.	Control group with other interventions
Sobieraj et al., 2009	Canadian Journal of Nursing Research Archive	The effect of music on parental participation during pediatric laceration repair.	Insufficient data
Sundar et al., 2016	Clinical pediatrics	Live Music Therapy as an Active Focus of Attention for Pain and Behavioral Symptoms of Distress During Pediatric Immunization	Insufficient data
Uggla et al., 2018	Acta paediatrica	Music therapy supported the health-related quality of life for children undergoing haematopoietic stem cell transplants	Insufficient data
Van Dokkum et al., 2020	Frontiers in Pediatrics	Feasibility of Live-Performed Music Therapy for Extremely and Very Preterm Infants in a Tertiary NICU	Not RCT
Viana et al., 2020	Rev Rene	Pain in full term newborns submitted to music and swaddling during venipunctures	Insufficient data
Whitehead-Pleaux et al., 2006	Journal of music therapy	The effects of music therapy on pediatric patients' pain and anxiety during donor site dressing change.	Insufficient data
Whitehead-Pleaux et al., 2007	Journal of music therapy	Exploring the effects of music therapy on pediatric pain: Phase 1	Not RCT

Supplementary File S2. Quality of the included studies:

(1) Risk of bias graph; (2) Risk of bias summary.

Fig S1.
Risk of bias graph: review authors' judgements about each risk of bias item presented as percentages across all included studies.

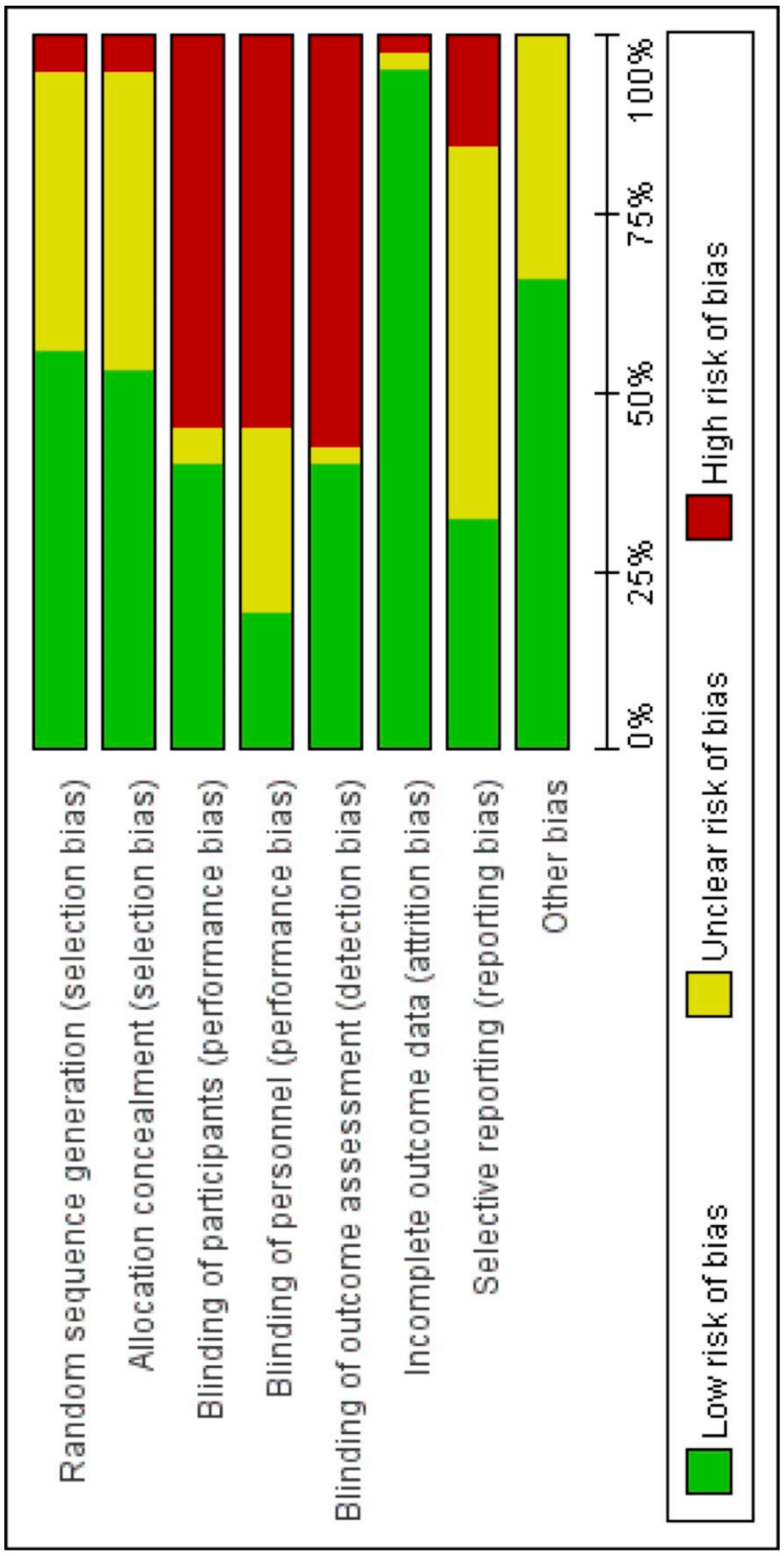

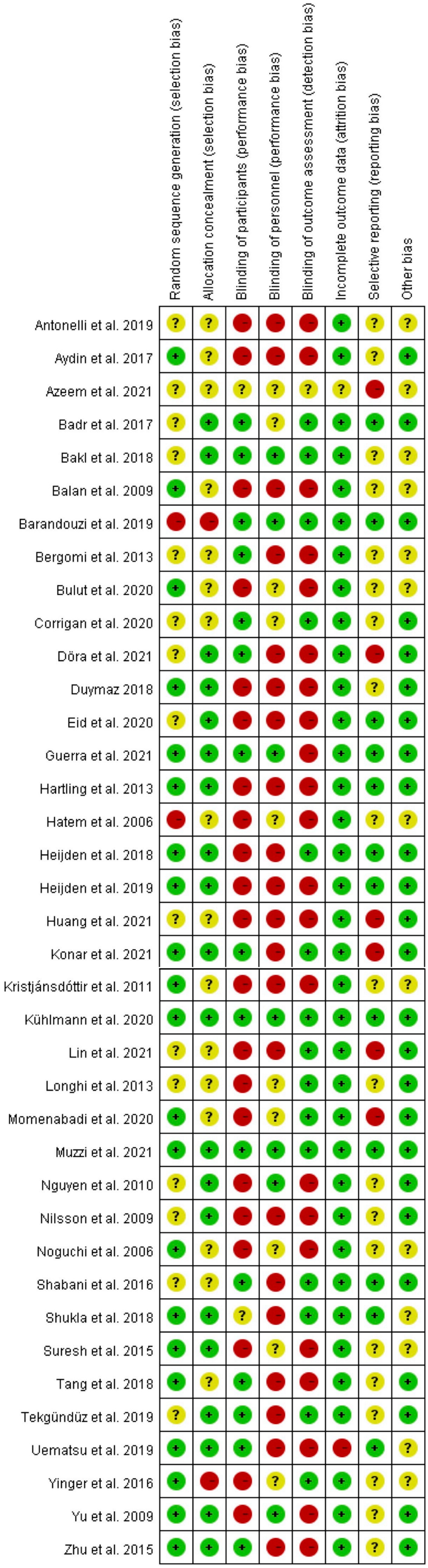

Fig S2.
Risk of bias summary: review authors' judgements about each risk of bias item for each included study.
